# Endometrial Dysfunction in Women with Ovarian and Uterine Tumors: What Is Known and What Should Be Learned?

**DOI:** 10.3390/ijms27052376

**Published:** 2026-03-04

**Authors:** Liudmila M. Mikhaleva, Mekan R. Orazov, Evgeny D. Dolgov, Sergey A. Mikhalev, Zarina V. Gioeva, Nikolay K. Shakhpazyan, Valentina V. Pechnikova, Mikhail Y. Gushchin

**Affiliations:** 1 Scientific Research Institute of Human Morphology Named After Academician A.P. Avtsyn of the Federal State Budgetary Scientific Institution “Russian Scientific Center of Surgery Named After Academician B.V. Petrovsky”, 117418 Moscow, Russia; mikhalevalm@yandex.ru (L.M.M.); 1586dolgde@gmail.com (E.D.D.); nshakhpazyan@gmail.com (N.K.S.); valiagtx@yandex.ru (V.V.P.); guschin.michail@yandex.ru (M.Y.G.); 2 Department of Obstetrics and Gynecology, Federal State Autonomous Educational Institution of Higher Education «Peoples’ Friendship University of Russia», 117198 Moscow, Russia; orazov-mr@rudn.ru; 3 Federal State Autonomous Educational Institution of Higher Education “N.I. Pirogov Russian National Research Medical University” of the Ministry of Health of the Russian Federation, 117997 Moscow, Russia; mikhalevsa1@zdrav.mos.ru; 4 Savelyeva Moscow City Clinical Hospital No. 31, 119415 Moscow, Russia

**Keywords:** endometrial dysfunction, implantation failure, reproductive failure, benign ovarian tumors, serous cystadenoma, mucinous cystadenoma, uterine fibroids

## Abstract

Multimorbidity is a key global trend across healthcare fields, including gynecology. It is strongly associated with an overall poorer health status. Statistics indicate that in the 21st century most women experience at least one gynecological disease. Meanwhile, there is a consistent increase in the prevalence of obesity associated with chronic inflammation and hyperestrogenism. Alongside other factors, it leads to a growing prevalence of hyperproliferative diseases of the female reproductive system (FRS), encompassing both benign and malignant conditions. While advanced-stage malignant tumors can be linked to missed detection and wrong checkup strategies, benign neoplasms can compromise the ovarian reserve and thus cause major concerns. The prevailing benign FRS tumors are uterine fibroids (UFs) and benign ovarian tumors (BOTs), including serous and mucinous cystadenomas. It appears that an increase in certain benign FRS tumors is occurring in parallel with a rise in infertility (especially “unexplained infertility”) and reproduction failures, potentially associated with endometrial dysfunction. Thus, the endometrium is currently considered a critical area of research due to its vital role as the site of blastocyst adhesion and implantation, especially in patients with comorbidities. In this context, this article highlights the significance and pathophysiological characteristics of UFs and BOTs and their impact on defective endometrial receptivity.

## 1. Benign Ovarian and Uterine Tumors: The Significance, Trends, and Global Burden

At the turn of the 20th and 21st centuries, the advanced technologies, automation, and algorithmization of diagnostic and therapeutic processes have driven a profound evolution in medical science. Nevertheless, the prevalence of multimorbidity is demonstrating continuous growth globally. And this ongoing trend is clearly observed in gynecology. The global situation regarding comorbidities is a growing and progressively severe public health challenge worldwide (for instance, in Europe, overweight and obesity affect almost 60% of adults and almost a quarter are living with obesity). An essential component of multimorbidity involves an increasing incidence of proliferative diseases, including both benign and malignant conditions [1]. A study by Japanese researchers (2024) revealed significant increases in gynecological cancer morbidity and mortality rates in Japan from 1980 to 2019, with ovarian cancer incidence tripling and uterine corpus (endometrial) cancer incidence increasing eightfold [2].

However, from the perspective of preventive medicine, the malignant transformation of benign lesions stems from clinician-related issues (missed detection) or incorrect female screening and checkup strategy. For this reason, it seems important to carry out an in-depth analysis of the most common benign proliferative diseases of the female reproductive system. As highlighted in the study of Wijeratne D. et al. [3], benign gynecological conditions (BGCs) are major causes of morbidity for women worldwide. The authors demonstrated that for women of reproductive age (aged 15 years and over) in high-income countries (HICs), 3.94% of all years lost to disability (YLDs) were due to BGC. In low- and middle-income countries (LMICs), this rate was even higher: 5.35% of all YLDs were related to BGCs. Thus, uterine leiomyomas (ULs), also known as uterine fibroids (UFs) or myomas, and benign ovarian tumors are increasingly recognized as the most prevalent benign proliferative conditions affecting the female reproductive system. In this regard, female infertility and implantation failures represent significant health issues that often coincide with other medical conditions, reflecting the current global trends in the prevalence of comorbidities. A recent analysis (2025) based on the global burden of disease study demonstrates that the worldwide prevalence of female infertility has substantially increased between 1990 and 2021: the age-standardized prevalence rate (ASPR) was recorded at 2764.62 per 100,000 individuals [4]. It should be borne in mind that in in vitro fertilization and embryo transfer (IVF-ET), implantation occurs in only 25% to 30% of transferred embryos, while achieving a 90% implantation success rate often requires at least three euploid embryos [5]. Furthermore, “the problem resides in the problem,” when it comes to recurrent implantation failures (RIFs) experienced by an estimated portion of 10–15% of IVF patients [6]. The modern understanding and etiological paradigms for RIFs are centered on reconciling embryo quality and functional endometrial disruption. Previously, it was considered that only poor-quality oocytes/blastocytes were “responsible” for reproductive losses, but it is currently accepted that the endometrium is not a passive partner to the active embryo in the pathogenesis of such failures. In some patients, essential components of RIF may involve a pathogenetic continuum, combining endometrial and embryo quality, though in other cases isolated endometrial dysfunction can affect implantation [7]. Thus, now it appears that gynecological comorbidities such as benign ovarian tumors and UFs may play an active role in the pathogenesis of endometrium-associated reproductive failures.

In summary, the objective of this review article is to analyze the pathogenesis of the most common benign conditions of the female reproductive system and to identify their potential molecular and biological associations and relationships with endometrial dysfunction.

## 2. Benign Ovarian Epithelial Tumors: Definition and Classification

Benign ovarian masses are, by classical definition, considered hyperproliferative diseases that do not exhibit the characteristics of malignancy; specifically, they lack features like oncogenic proliferation, the presence of typical cells, invasive potential, and the ability to metastasize [8]. Currently, ovarian tumors are categorized according to the 2020 WHO Classification of Female Genital Tumors [9]. However, the classification does not contain a specific section for the benign neoplasms analyzed in our article. The classification divides tumors into groups that include benign, borderline, and malignant (Table 1).

Overall, benign ovarian masses encompass a diverse range of tumor histotypes and tumor-like lesions, making it challenging to present their exact structure due to the variability in epidemiological data. At the same time, evidence consistently indicates that benign ovarian masses account for approximately 80% of all ovarian neoplasms in patients of reproductive age, while only about 20% are malignant ovarian carcinomas [10]. Recent research in India, involving 110 cases of ovarian neoplasms, found that epithelial tumors were the most common type. Among these epithelial tumors, serous and mucinous cystadenomas were the predominant benign subtypes, observed in 38% and 7.2% of cases, respectively [11].

In view of the above findings, this article will highlight the complex pathogenetic interplay between the endometrium and benign epithelial tumors of the ovaries (specifically, serous and mucinous cystadenomas) recognized as the most common ovarian neoplasms.

### 2.1. Pathogenesis Pathways

#### 2.1.1. Common Pathogenetic Mechanisms

While the exact mechanisms behind benign ovarian tumors are still being researched, the long-standing controversy has centered on various theories of their etiopathogenetic aspects. Ovarian epithelial neoplasms can be categorized as primary (originating within the ovary) or secondary (originating outside the ovary). The distinction represents a significant subject of ongoing debate. The theory of primary origin of ovarian epithelial tumors was proposed in 1995, when Scully, R. E. revealed that most ovarian serous tumors develop in the ovarian stroma without being connected to surface epithelium [12]. However, recent studies have strongly suggested that the fallopian tube epithelium is a potential primary site of origin of ovarian epithelial neoplasms, since it exhibits active proliferation and can invade the ovaries [13,14].

In this regard, it is worth noting a recent histopathological study of Silva E. G. et al. [15] analyzing 300 ovarian epithelial neoplasms. The authors determined major proliferative sources of ovarian tumors (including malignant neoplasms). These key sources are described below:

- Ovarian inclusion cysts: retention structures lined with epithelial cells and located within the ovarian cortex.
- Mesenchymal–epithelial transition: endosalpingiosis (the invasion and presence of fallopian tube-like columnar epithelium on the surface of the ovary; these ectopic epithelial cells can subsequently form cysts), inverted macropapillae (papillar structures covered by epithelial and invaginated into the adjacent ovarian stroma); polyploid giant cancer cells (PGCCs).
- Simple cysts: retention structures that form through the invagination of the ovarian surface mesothelium and show no signs of tubular metaplasia.

The authors performed detailed histopathological analysis to determine an association of potential precursors with different types of ovarian neoplasms (Table 2) [15].

**Figure 1:**
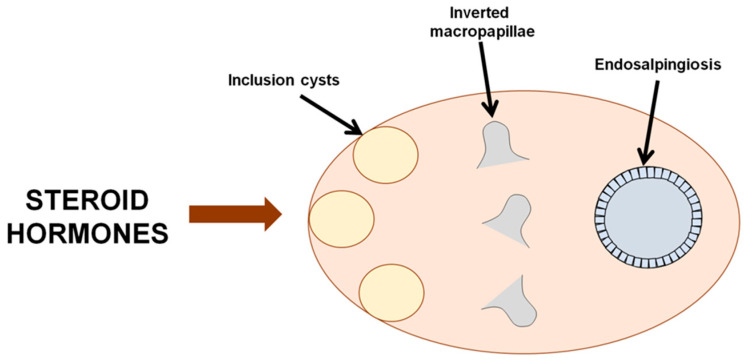
Potential sources of ovarian epithelial neoplasms [15].

Thus, the evidence indicates that benign ovarian neoplasms (specifically, serous adenomas and adenofibromas) arise from several sources: inclusion cysts, fragments of the tubular epithelium (endosalpingiosis), and inverted macropapillae of the ovarian stroma (Figure 1).

#### 2.1.2. Molecular Alterations (Gene Mutations, Epigenetic Changes)

At the same time, it is important to synthesize these complex morphological and histological findings with the underlying cellular and molecular changes that trigger and drive abnormal proliferation in ovarian structures. The present article points to the role of specific genetic aberrations in different ovarian neoplasm subtypes, particularly highlighting mutations for carcinomas. The evidence suggests that ovarian epithelial tumors exhibit a diverse genomic landscape, with the most common and significant alterations being mutations in the *BRCA1* and *BRCA2* genes. These *BRCA1* and *BRCA2* mutations are strongly associated with serous and mucinous carcinomas, though their presence was revealed across a range of other epithelial types, including endometrioid neoplasms [16]. It is noteworthy that mutations in the *BRCA1* and *BRCA2* genes are recognized as major predictors of oncogenic transition, but they are generally not used as prognostic biomarkers for benign neoplasms. The role of these mutations in benign cystadenomas or borderline tumors as precursors in BRCA-associated ovarian carcinomas is a subject of ongoing debate. Currently, evidence-based information in this scientific field is missing, and it is possible that future studies will confirm that these genes are not primary drivers in the development of benign epithelial tumors.

Research has also confirmed that the most frequent molecular alterations in all benign mucinous ovarian neoplasms are *KRAS* mutation and cyclin-dependent kinase inhibitor 2A *(CDKN2A)* inactivation [16]. The additional evidence supporting this finding is derived from the study of Mackenzie R. et al. [17]. It demonstrated that *KRAS* and *CDKN2A* mutations were the most frequently observed alterations among patients with mucinous ovarian tumors (MOTs), including borderline and cancerous neoplasms. In the same group of patients, other genetic aberrations were detected, such as mutations in *TP53*, *PIK3CA*, *PTEN*, *BRAF*, etc. However, benign serous cystadenomas (BSCs) exhibit a different genetic pattern, as they do not show *KRAS* or *BRAF* mutations. Serous cystadenomas appear to progress to ovarian serous borderline tumors (OSBTs) due to *BRAF* mutation, while OSBTs progress to ovarian low-grade serous carcinoma (OLGSCa) due to *KRAS* mutation [18].

On the other hand, immunohistochemically, PAX8 and WT1 were positive in specimens of ovarian serous cystadenoma [19]. It is also worth noting the results of recent research which determined the key differential diagnosis biomarkers that can be used to distinguish high-grade serous ovarian cancer (HGSOC) cases from benign ovarian tumors. In this regard, comparing gene promoter methylation in ovarian cancer (OC) cases with benign tissues, three *HOX*-related gene promoters had significant increases (*p* < 0.04) in methylation frequency in OC. Six notch pathway-related (*NOTCH1*, *NOTCH2*, *NOTCH3*, *NOTCH4*, *DLL1*, and *HES1*) and two Wnt pathway-related (*CTNNB1* and *FBXW7*) genes showed significantly lower expression in OC cases compared to benign tissues (*p* = 0.036 for *NOTCH1* and *p* < 0.001 for all other genes) [20].

In general, the pathogenesis of benign ovarian epithelial tumors and associated hyperproliferation in the ovarian structures is driven by specific genetic changes. At the same time, there are significant differences in genetic patterns of serous and mucinous ovarian tumors. Mucinous ovarian tumors are characterized by frequent mutations in oncogenes, and due to the presence of specific invasive patterns they show an aggressive course and a high risk of progression to borderline and malignant tumors. Ovarian serous cystadenomas exhibit a different genetic profile and generally have a favorable prognosis.

Unfortunately, currently, it is not possible to definitively determine the role of various genetic predictors in benign ovarian neoplasms, as studies in this area face significant methodological challenges, including small sample sizes and observational or retrospective designs and often rely on indirect statistical conclusions. Thus, multiple issues regarding this topic remain to be addressed.

#### 2.1.3. Dysfunction of the Immune System

Lu Y. et al. in their recent research [21] contextualized the role of the immune system in maintaining ovarian homeostasis and elucidated the casual links between multiple immune cell phenotypes and benign ovarian-related diseases. As demonstrated, CD4+ CD8dim T cells, CD24 on CD24+ CD27+ B cells, and IgD on IgD+ CD38dim B cells act as protective factors against benign neoplasms of the ovary.

The findings from their research indicate that dysfunction in immune patterns may lead to the abnormal proliferation of ovarian tissues and the invasion of ectopic tubular epithelial cells, ultimately contributing to the formation of ovarian neoplasms. Additionally, the study revealed that B-cell activating factor receptor (BAFF-R) was associated with an increased risk of malignant ovarian neoplasm, while CD25 on activated CD4 regulatory T cells and CD45 on lymphocytes were associated with a decreased risk of malignant neoplasms [21]. Thus, the research specifically confirmed a causal link between certain immune cell signatures and various ovarian conditions, including those involving abnormal cell proliferation (benign and malignant ovarian tumors). In summary, the three key mechanisms involved in the pathogenetic interplay of benign ovarian epithelial tumors, as determined by the study, are genetic aberrations; the disruption of the stable internal conditions of the local immune system; and abnormal proliferation in the deeper layers of the ovarian tissue (Table 3).

## 3. Uterine Leiomyoma: Epidemiology, Classification and Pathogenesis

Uterine leiomyoma (UL), also known as uterine fibroids (UFs), is the most common benign monoclonal well-circumscribed tumor of the uterine body and cervix, originating from myometrial smooth muscle cells (progenitor/stem cells) [22,23]. Studies suggest that UFs occur in about 25–50% of women worldwide [24].

Uterine fibroids pose a significant global public health challenge due to their high prevalence among women of reproductive age. According to the comparative cost analysis of Hazimeh, D. et al. [25] the number of US women with uterine fibroids increased by 10.6% from 2010 to 2022. Over this period, the overall economic burden of uterine fibroids increased to USD 42.2 billion compared to USD 34.4 billion in 2010. Recently, a clear link has been established between clinical profile, medical history, and a high risk of developing uterine leiomyoma (UL). The recent research by Munro, M. G. et al. [26] has highlighted the following key risk factors for UL:

- Later reproductive years.
- Racial associations: Black women have a significantly higher risk of uterine leiomyomas compared to white women. Additionally, black women are more likely to develop multiple fibroids; for instance, they have approximately 1.5-fold greater propensity of having seven or more leiomyomas compared to white women.
- Early menarche.
- Nulliparity.
- Genetic predisposition.
- Obesity.
- Vitamin D deficiency.

**Figure 2:**
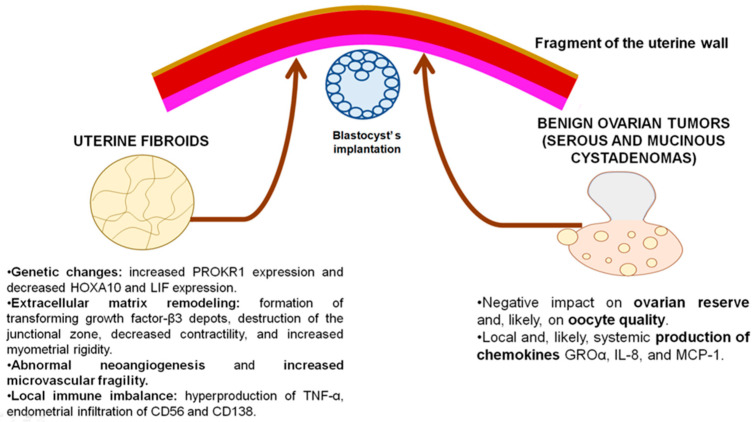
Established and potential pathogenetic associations between benign cystadenomas/uterine leiomyomas and endometrial dysfunction.

The modern anatomically and topographically based system classifies uterine fibroids by their location relative to the layers of the uterus (as subserous, intramural, or submucous) [27]. At present, clinical settings widely use the uterine leiomyoma classification system developed by the International Federation of Gynecology and Obstetrics (FIGO) in 2011. This system, known as the FIGO Classification of Uterine Fibroid Locations, standardized the terminology for describing the location of fibroids relative to the uterine wall and cavity: from Type 0 for “pedunculated intracavitary” fibroids to Type 7 for “pedunculated subserosal” and Type 8 for other types of fibroids, e.g., cervical (Figure 2) [28,29]. In 2019, the World Health Organization identified several common histological types of benign uterine leiomyoma, including cellular, mitotically active, lipomatous leiomyoma, and other variants [30].

### 3.1. Pathogenesis Pathways

It is currently a widely accepted consensus that UFs are hormone-dependent tumors, and evidence suggests that estrogens and progesterone play a causative role in the proliferative activity of these tumors. Furthermore, the modern understanding of UFs is a complex pathogenetic concept involving a broad array of molecular interactions and an interplay of epigenetic and genetic factors. The pathogenetic concept of UFs, including the key patterns, is presented below:

### 3.2. Genetic Factors

A 2021 study by Finnish researchers aimed to characterize the genetic drivers of uterine leiomyomas in a large sample of over 700 patients. The structure of genetic aberrations was described in various genes, while the predominant mutations included *MED12* (77.4%), *HMGA2* (7.3%), and *HMGA1* (3.0%) [31]. Similar findings were reported in research published in 2024 which included 1353 patients and 1872 fibroid tumors. Of the total number of tumors analyzed, 1045 (55.8%) harbored a *MED12* mutation. In addition, the evidence suggested that *MED12* gene mutations are linked to an increase in the potential mitotic activity of myocytes. *MED12* mutation acts by increasing levels of protein kinase B (AKT) and disrupting cyclin C-CDK8/19 kinase activity [32].

### 3.3. Steroid Receptor Imbalance

Accumulating evidence suggests that progesterone signaling promotes the activation of the mitogenic potential of smooth muscle cells by increasing the expression of proliferating cell nuclear antigen (PCNA) and WNT ligands. In addition, progesterone has been shown to suppress apoptosis in fibroid cells by increasing the expression of Bcl-2 protein [33]. There is also evidence that estrogens improve the responsiveness of the cells to progesterone, which in turn upregulates the expression of PCNA, and increase the number of progesterone receptors (PRs), thus potentiating the key effects of progesterone [34].

### 3.4. Pro-Inflammatory Factors

As demonstrated in the studies, the expression levels of pro-inflammatory and inflammatory cytokines, such as IL-1, IL-6, IL-10, TNF-α, and TGF-β, are upregulated in UFs, promoting their growth. These cytokines play a crucial role by activating local growth factors and interacting with extracellular matrix components [35]. It is worth noting that most pro-inflammatory markers, particularly a broad panel of cytokines, are secreted by immunocompetent cells. Recent research has revealed a distinct peripheral immune landscape in the presence of UFs. The study by Li, P. X. et al. [36] demonstrated systemic immune dysregulation in UF patients, especially within helper T (Th) cell compartments, characterized by elevated frequencies of functional Th and Th17 cells, alongside reduced proportions of senescent Th, T follicular helper 1 (Tfh1), and peripheral Th (Tph) cells. In parallel, a significant expansion of the B cell compartment was observed, marked by increased total B cells, naïve B cells, immature regulatory B cells (Breg), and transformed B cells. These findings have shown that the local inflammatory profile driven by an imbalance of immunocompetent cells is a key component in the pathogenesis of UFs.

Thus, the modern understanding of the pathogenesis of UFs points to a complex interplay of multiple factors, including genetic, steroid receptor, and local molecular biological processes. Overall, these pathogenetic patterns involve various mechanisms, affecting the endometrium (as described in more detail below), and are not just limited by the remodeling of the myometrium.

## 4. Endometrial Dysfunction as a Reflection of Benign Tumors in the Ovaries and Uterine Body

### 4.1. Benign Ovarian Cystadenomas

#### Evidence-Based Relationships and Potential Pathogenesis Pathways

From this perspective, it seems important to stress that the pathogenesis of benign ovarian epithelial tumors (specifically, serous and mucinous cystadenomas) is linked to genetically induced hyperproliferation of various ovarian structures. However, only limited data describing the impact of benign ovarian cystadenomas on endometrial receptivity can be found in the scientific literature. In this regard, it is worth noting the study of Tumasyan, E.A. et al. [37] which included 129 patients (77 with epithelial ovarian tumors (EOTs) and 52 with mature teratomas). The study demonstrated that ovarian epithelial tumors can negatively impact the morphological and functional features of the endometrium, primarily by reducing the number of mature pinopodes and decreasing the expression levels of progesterone receptors (PRs), both of which are crucial for successful embryo implantation. The combined effect of these factors disrupts the “implantation window,” making the endometrium less conducive to pregnancy. After surgical treatment, the endometrial steroid receptive status significantly improved in the group of patients with ovarian epithelial tumors. In patients with mature teratomas, the number of mature pinopodes and steroid receptors at the pre-operative stage were within the normal physiological range, but after the surgical treatment their numbers decreased. Thus, unintentional intra-operative removal or damage of normal ovarian tissue can negatively impact endometrial implantation patterns. Thus, the evidence indicates that surgical damage to intact ovarian tissue can lead to endometrial dysfunction associated with a steroid hormone imbalance and potential alterations in their respective receptor functions. However, the issue regarding the potential impact of non-operated benign ovarian tumors on endometrial status is a subject of ongoing debate and controversy.

There is also evidence that a local and systemic pro-inflammatory microenvironment is involved in the pathogenesis and progression of benign ovarian tumors. In this context, Kristjánsdóttir, B. et al. [38] found significantly elevated levels of pro-inflammatory chemokines (GROα, IL-8, MCP-1) in the ovarian cyst fluid samples of patients with benign ovarian tumors. For this reason, ovarian tumors, even benign ones, can create a pro-inflammatory environment that negatively impacts endometrial structure and receptivity during the implantation window. This inflammatory state exerts both local and distant effects, disrupting steroid hormone production and affecting the endometrium. However, precise confirmation of this impact requires further laboratory research and clinical studies.

In this context, it is important to highlight the results of research by Shandley Lisa, M. et al. [39] which included 1537 women of reproductive age. It showed that infertility was more common in women who underwent ovarian cyst surgery than those without surgery (median-adjusted hazard ratio 2.41, 95% simulation interval 1.03–6.78). Paradoxically, the estimated geometric mean anti-Müllerian hormone (AMH) levels of those who reported a history of ovarian cyst surgery were 1.08 (95% CI: 0.57–2.05) times those of women who reported no history of surgery. These findings suggest that damage to intact ovarian tissue during surgery may reduce ovarian reserve. Furthermore, it can negatively impact the endometrium.

There is evidence that ovarian tumors often affect the ovarian reserve. Even benign ovarian tumors have a negative impact on the antral follicular count (AFC) and levels of anti-Mullerian hormone (AMH). The study by Bareghamyan, H. et al. [40] revealed a significant decrease in AMH and AFC levels among participants with benign ovarian cystadenomas. The findings suggest that the presence of such benign lesions may lead to infertility and reproduction failures in this patient group. In conclusion, the pathogenesis of infertility in patients with benign ovarian tumors is a complex issue that is linked not only to the direct or indirect impact of the tumors themselves, but also significantly associated with the potential damage to healthy ovarian tissues during their surgical removal. This damage can disrupt steroid receptor factors, leading to implantation failures.

### 4.2. Uterine Fibroids

#### Anatomical Factors and Steroid Hormone Dysregulation

Currently, the pathogenetic association between UFs and endometrial dysfunction remains a complex and controversial issue. It appears that due to the adjacent location of two anatomical compartments (the myometrium and endometrium), the key molecular and biological disruptions occurring in and around uterine leiomyomas have a negative impact on the endometrium. In this regard, the study by Kudimova, M.A. et al. [41] demonstrated that uterine leiomyomas were associated with endometrial dysfunction, which disrupts the formation of the implantation window. A key aspect of this disruption involves the impaired development and reduced number of pinopodes within the endometrium. In addition, inflammatory tissue infiltration with CD56 and CD138 cells was found in patients with uterine leiomyomas. These patients also showed elevated levels of expression of both estrogen receptors (ERs) and progesterone receptors (PRs) in the endometrial glands. Thus, it appears that uterine fibroids (UFs) can negatively impact the endometrium, with the severity largely dependent on their anatomical location. It is apparent that submucosal leiomyomas exert the most significant effects on the development of endometrial dysfunction, while subserosal fibroids are less likely to impact endometrial homeostasis. At the same time, intramural leiomyomas can negatively affect the endometrium if located in proximity to the junctional zone and distort the uterine cavity [42]. Thus, research has confirmed a direct relationship between the anatomical location of myoma knots and the risk of reproductive failures. However, it appears that heightened interest exists in the potential pathogenetic interactions between UFs and the intact endometrium. For this reason, the authors provide a summary of the potential mechanisms associated with the negative impact of UFs on the endometrium leading to implantation failure.

### 4.3. Genetic Factors

Recent research by Özbey, G. et al. [43] has demonstrated that the expression levels of endometrial receptivity-related genes were significantly altered in patients diagnosed with myoma uteri. Gene expression levels of endometrial *PROKR1* were statistically significantly increased and gene expression levels of *PROK1*, *PROKR2*, and *HOXA10* were found to be statistically significantly decreased compared to the controls. These findings may explain certain aspects of implantation failure in these patients. Notwithstanding this, the most important myoma-induced changes in the endometrium involve altered *HOXA10* and leukemia inhibitory factor (*LIF*) expression. Recent studies suggest that *HOXA10* and *LIF* play an essential role in the process of blastocyst adhesion and nidation: *HOXA10* is involved in maintaining the endometrial receptivity required for the embryo to attach, while *LIF* acts locally in the endometrium as a crucial molecular signal for successful implantation [44,45]. Other studies also confirm that a decrease in *HOXA10* and *LIF* expression in patients with uterine leiomyoma contributes substantially to endometrial dysfunction [46,47]. It is also worth noting the results of recent research by Hosseini, M. et al. [48], which has identified three shared key genes (*EDNRB*, *BIRC3* and *TRPC6*) between UFs and repeated implantation failures (RIFs). Targeting these genes could lead to treatments addressing the root causes of UFs and RIF [48].

#### 4.3.1. Extracellular Matrix Remodeling

Research has demonstrated that one of the most common characteristics of UFs is the excessive production of extracellular matrix (ECM) components, including collagens, glycosaminoglycans, fibronectin, and laminins. These components may serve as “biological reservoirs” of profibrotic growth factors such as transforming growth factor β3 and modulate their molecular and biological effects. This biological substrate provokes bone morphogenetic protein-2 (BMP-2) resistance, and lower levels of BMP2 are linked to decreased endometrial stromal cell expressions of *HOXA10* and *LIF*. In addition, ECM remodeling leads to mechano-transduction changes due to the destruction of the junctional zone or the endometrial–myometrial interface that result in a decrease in uterine wall contractility and increased myometrial rigidity. Ultimately, this series of changes is associated with defective myometrial decidualization, impaired implantation, and an increased risk of reproduction failures [49].

#### 4.3.2. Abnormal Angiogenesis

Research findings have demonstrated that the presence of UFs upregulated the expression of endometrial angiogenic factors, including vascular endothelial cell growth factor (VEGF) and adrenomedullin (ADM). As shown, ADM is involved in the regulation of the endogenous vasodilator nitric oxide. Overexpression of VEGF and adrenomedullin (ADM) is believed to contribute to abnormal endometrial angiogenesis and increased vascular fragility in the endometrial compartment and subsequent endometrial dysfunction [50].

#### 4.3.3. Local Immune Imbalance

The evidence suggests that UFs stimulate the formation of a specific “pro-inflammatory rim” involving the endometrium. In this context, recent research has demonstrated that the endometrium of patients affected by UFs displayw elevated tumor necrosis alpha (TNF-α) expression levels which contribute to a higher risk of reproductive failures [51]. The impaired secretion of anti-inflammatory cytokines in the endometrium of women with UFs plays an important role in local immune imbalance. Thus, UFs can induce a decrease in interleukin-11 (IL-11) expression, which is linked to disrupted endometrial decidualization. In turn, the disruption in decidualization can interfere with adequate implantation of the gestational sac [52].

## 5. Conclusions

In summary, benign ovarian tumors and uterine fibroids remain the most significant gynecological proliferative diseases. Our review summarizes recent research advances on the pathogenesis of these gynecological disorders and highlights the key roles of genetic aberrations and local immune imbalance in the development of benign ovarian epithelial tumors, such as cystadenomas. At the same time, uterine fibroids (UFs) are characterized by a complex and broader pathogenetic profile, including genetic changes, local steroid receptor dysregulation, and an imbalance of pro- and anti-inflammatory cytokines. This article also elucidates the pathogenetic associations between UFs, benign ovarian epithelial tumors, and endometrial dysfunction. In the scientific community, there is widespread agreement that UFs cause significant local molecular changes and negatively impact the endometrium, while endometrial dysfunction induced by benign ovarian tumors is still a subject of ongoing debate and thus requires further research (Figure 2). Despite the current advances in research, there are significant challenges in establishing consistent patterns of endometrial dysfunction associated with UFs or BOTs. The critical challenge is linked to conducting a broad comprehensive morphopathological analysis on large sample sizes. Moreover, most cited studies are retrospective or have methodological limitations. In this context, the goal of our review article was to reflect on the potential multifactorial mechanisms of endometrial dysfunction. We hope that this article will provide a framework for further evidence-based studies by offering a foundation for validating/updating current findings, potential avenues for discovering new mechanisms underlying this condition, and a new perspective on this issue.

## Figures and Tables

**Table 1:** 2020 WHO classification of ovarian tumors.

2020 WHO Classification of Ovarian Tumors.	
Serous tumors	Mesenchymal tumors
Mucinous tumors	Mixed epithelial and mesenchymal tumors
Endometrioid tumors	Sex cord–stromal tumors
Clear cell tumors	Germ cell tumors
Seromucinous tumors	Miscellaneous tumors
Brenner tumors	Tumor-like lesions
Other carcinomas	Metastases to the ovary

**Table 2:** Major precursors of ovarian epithelial tumors [15].

	Inclusion Cysts	Endosalpingiosis	Inverted Macropapillae	Polyploid Giant Cells	Simple Cysts
	Serous Neoplasms				
Serous cystadenoma, adenofibroma	100%		14%	-	5%
Low-grade serous carcinoma	72%		30%	-	10%
High-grade serous carcinoma	11%		4.5%	20%	42%

**Table 3:** Key pathogenetic mechanisms associated with benign ovarian tumors.

Key Mechanisms	Comments
Proliferation in the ovarian and extra-ovarian structures	Ovarian structures: inclusion cysts, inverted macropapillae of the ovarian stroma. Extra-ovarian structures: heterotopic tubular epithelium (endosalpingiosis).
Genetic aberrations	Aberrations common for all epithelial tumors: *BRCA 1/2*. Markers expressed in benign serous tumors: *PAX8*, *WT*. Specific alterations for benign mucinous tumors: *KRAS* mutations and *CDKN2A* aberrations.
Immune cell signatures	Associated with a decreased risk of benign ovarian neoplasms: CD4+ CD8dim T cells, CD24 on CD24+ CD27+ B cells, and IgD on IgD+ CD38dim B cells.

## Data Availability

No new data were created or analyzed in this study.

## Abbreviations

The following abbreviations are used in this manuscript:

FRS	Female reproductive system
FIGO	Federation of Gynecology and Obstetrics
ULs	Uterine leiomyomas
UFs	Uterine fibroids
BOTs	Benign ovarian tumors
ECM	Extracellular matrix
PGCCs	Polyploid giant cancer cells
